# 6β-Hydroxytestosterone, a metabolite of testosterone generated by CYP1B1, contributes to vascular changes in angiotensin II-induced hypertension in male mice

**DOI:** 10.1186/s13293-019-0280-4

**Published:** 2020-01-16

**Authors:** Ajeeth K. Pingili, Brett L. Jennings, Kamalika Mukherjee, Wadah Akroush, Frank J. Gonzalez, Kafait U. Malik

**Affiliations:** 1 Department of Pharmacology, College of Medicine, University of Tennessee Health Science Center, 71 S. Manassas TSRB, Memphis, TN 38103 USA; 2 Laboratory of Metabolism, National Cancer Institute, Bethesda, MD 20892 USA

**Keywords:** Angiotensin II, Hypertension, CYP1B1, Testosterone metabolite 6β-hydroxytestosterone, Vascular reactivity, Endothelial dysfunction, Vascular hypertrophy, Fibrosis, Flutamide

## Abstract

**Background:**

Previously, we showed that 6β-hydroxytestosterone (6β-OHT), a cytochrome P450 1B1 (CYP1B1)-derived metabolite of testosterone, contributes to angiotensin II (Ang II)-induced hypertension in male mice. This study was conducted to test the hypothesis that 6β-OHT contributes to increased vascular reactivity, endothelial dysfunction, vascular hypertrophy, and reactive oxygen species production associated with Ang II-induced hypertension.

**Methods:**

Eight- to 10-week-old intact or castrated C57BL/6 J (*Cyp1b1*^*+/+*^ and *Cyp1b1*^−/−^) mice were anesthetized for implantation of a micro-osmotic pump which delivered Ang II (700 ng/kg/day) or saline for 14 days. Mice were injected with 6β-OHT (15 μg/g b.w every third day), flutamide (8 mg/kg every day), or its vehicle. Blood pressure was measured via tail-cuff. Vascular reactivity, endothelial-dependent and endothelial-independent vasodilation, media to lumen ratio, fibrosis by collagen deposition, and reactive oxygen species production by dihydroethidium staining were determined in the isolated thoracic aorta.

**Results:**

The response of thoracic aorta to phenylephrine and endothelin-1 was increased in Ang II-infused *Cyp1b1*^*+/+*^ mice compared to intact *Cyp1b1*^*−/−*^ or castrated *Cyp1b1*^*+/+*^ and *Cyp1b1*^*−/−*^ mice; these effects of Ang II were restored by treatment with 6β-OHT. Ang II infusion caused endothelial dysfunction, as indicated by decreased relaxation of the aorta to acetylcholine in *Cyp1b1*^+/+^ but not *Cyp1b1*^−/−^ or castrated *Cyp1b1*^+/+^ and *Cyp1b1*^*−/−*^ mice. 6β-OHT did not alter Ang II-induced endothelial dysfunction in *Cyp1b1*^+/+^ mice but restored it in *Cyp1b1*^−/−^ or castrated *Cyp1b1*^+/+^ and *Cyp1b1*^*−/−*^ mice. Ang II infusion increased media to lumen ratio and caused fibrosis and reactive oxygen species production in the aorta of *Cyp1b1*^+/+^ mice. These effects were minimized in the aorta of *Cyp1b1*^−/−^ or castrated *Cyp1b1*^+/+^ and *Cyp1b1*^*−/−*^ mice and restored by treatment with 6β-OHT. Treatment with the androgen receptor antagonist flutamide reduced blood pressure and vascular hypertrophy in castrated Ang II-infused mice injected with 6β-OHT.

**Conclusions:**

6β-OHT is required for the action of Ang II to increase vascular reactivity and cause endothelial dysfunction, hypertrophy, and increase in oxygen radical production. The effect of 6β-OHT in mediating Ang II-induced hypertension and associated hypertrophy is dependent on the androgen receptor. Therefore, CYP1B1 could serve as a novel target for the development of therapeutics to treat vascular changes in hypertensive males.

## Introduction

Hypertension is the leading cause of cardiovascular diseases, renal dysfunction, and end-organ damage, and biological sex plays a significant role in the pathogenesis of hypertension and associated end-organ damage [1–4]. Sex differences in the development of hypertension and alterations in cardiovascular and renal function have been demonstrated in various experimental models of hypertension, which has been attributed to sex chromosomes and sex hormones [5–9]. Ang II increases blood pressure (BP) to a much higher level in males than in females, and it is reduced by castration in males, but enhanced by ovariectomy in females [10]. Previously, we demonstrated that the protective effect of 17-β estradiol against Ang II-induced hypertension and associated cardiovascular and renal pathophysiological changes are mediated most likely by its metabolite, 2-methoxyestradiol generated by CYP1B1 in female mice [11–13]. However, in contrast, Ang II-induced hypertension and cardiac and renal pathological changes that were minimized in castrated or *Cyp1b1*^*−/−*^ mice were restored by treatment with testosterone-CYP1B1 generated metabolite 6β-hydroxytestosterone (6β-OHT) [14–17].

In as much as 6β-OHT treatment alone did not produce any effect, we concluded that it acts as a permissive factor, in that it is required for the expression of these effects of Ang II [16–17]. Since Ang II causes vascular dysfunction, hypertrophy, fibrosis, and reactive oxygen species production (ROS) [18], we hypothesized that 6β-OHT mediates these vascular effects of Ang II in male mice. To test this hypothesis, we investigated the contribution of 6β-OHT to the effects of Ang II to increase vascular reactivity, endothelial dysfunction, hypertrophy, fibrosis, and ROS production in Ang II-induced hypertension in the thoracic aorta of castrated *Cyp1b1*^+/+^ and *Cyp1b1*^*−/−*^ mice that lack endogenous testosterone and 6β-OHT.

## Materials and methods

### Materials

Angiotensin II (Ang II) was purchased from Bachem (Torrance, CA), dihydroethidium (DHE) from Invitrogen (Carlsbad, CA), and 6β-hydroxytestosterone (6β-OHT) from Steraloids (Newport, RI). Phenylephrine, endothelin-1, acetylcholine, the Masson trichrome staining kit, and phosphate-buffered saline were purchased from Sigma (St. Louis, MO).

### Animals

All experiments were carried out according to protocols approved by the University of Tennessee Health Science Center Institutional Animal Care and Use Committee and in accordance with the National Institutes of Health Guide for the Care and Use of Laboratory Animals. C57BL/6J *Cyp1b1*^+/+^ male mice were purchased from Jackson Laboratory (Bar Harbor, ME), and *Cyp1b1*^−/−^ male mice from the C57BL/6J background were initially generated at the National Cancer Institute [19], and then bred at the University of Tennessee Health Science Center. The genotype of all *Cyp1b1*^*+/+*^ and *Cyp1b1*^−/−^ mice was routinely assessed by polymerase chain reaction (PCR) as described [19]. Eight- to 10-week-old male mice were acclimatized in restrainers for 1 week, and blood pressure was measured 2–3 times via tail-cuff. The animals were then anesthetized with a mixture of ketamine (87 mg/kg, i.p.) and xylazine (13 mg/kg, i.p.), and the micro-osmotic pumps (Alzet®; model 1002) were implanted subcutaneously to infuse Ang II (700 ng/kg/min) or saline (vehicle) for 14 days. BP was measured in the mice that were used to determine the contribution of 6β-OHT (15 μg/g, i.p. every third day) to Ang II-induced hypertension and associated cardiac pathogenesis [16]. In the present study, the following groups of these mice were used to assess aortic vascular reactivity, endothelial dysfunction, media to lumen ratio, fibrosis, and reactive oxygen species production:

- 6β-OHT: *Cyp1b1*^***+/+***^ and *Cyp1b1*^***−/−***^ mice were infused with either Ang II or vehicle for 14 days and injected with 6β-OHT (15 μg/g, i.p.) every third day.
- Castration: Eight-week-old *Cyp1b1*^***+/+***^ and *Cyp1b1*^*−/−*^ mice were castrated as described [2]. After a 7-day washout period for the depletion of residual testosterone, the mice were divided into two groups and infused with either vehicle or Ang II as described above.
- Castration+6β-OHT: Eight-week-old *Cyp1b1*^***+/+***^ and *Cyp1b1*^***−/−***^ mice were castrated and infused with either Ang II or vehicle for 14 days and injected with 6β-OHT every third day.

The following additional group of mice was used to perform experiments with the androgen receptor antagonist flutamide and its vehicle:

Castration+6β-OHT+ Flutamide: Eight-week-old *Cyp1b1*^−/−^ mice were castrated and infused with either Ang II or vehicle for 14 days, and then injected with the androgen receptor antagonist flutamide [9] (8 mg/kg i.p. daily) and 6β-OHT 15 μg/g i.p. every third day). Systolic blood pressure (SBP) was measured in these mice via tail cuff, and degree of thoracic aorta hypertrophy was determined as described below.

### Measurement of vascular reactivity

Following anesthesia as described above, the thoracic aortae were quickly dissected free, cleaned of surrounding tissue, and approximately 2-mm rings were mounted in a wire myograph system (Danish Myo Technology, Aarhus, Denmark; model 610M). Vascular reactivity was measured as described [11]. Cumulative concentration-response curves to phenylephrine (PE) and endothelin-1 (ET-1) of aortic rings were measured as the force of contraction in millinewton. Viability of the thoracic aorta was determined by measuring its contraction in response to KCl (60 mM) before and after exposure to PE and ET-1.

### Endothelium-dependent and endothelium-independent relaxation of the aorta

Endothelial function was assessed by measuring the magnitude of relaxation by increasing concentrations of acetylcholine (ACh) in the aortic rings pre-constricted maximally with PE (10^−5^ mol/L) as described [11]. Endothelium-independent vasodilation was studied by constricting the vessels with the concentration of PE that evoked a maximal response followed by the addition of increasing concentrations of sodium nitroprusside (SNP). Changes in the response of vessels to SNP were measured and presented as a percentage of the PE-induced constriction as described [11].

### Measurement of media/lumen ratio

Following anesthesia, the thoracic aortae were dissected free, cleaned of surrounding tissue, and frozen in optimal cutting temperature (OCT) compound (Sakura Finetek USA Inc., Torrance, CA). Aortic sections (5 μm) were stained with hematoxylin and eosin (H&E). Sections were viewed in a blinded manner using an Olympus® inverted system microscope (Olympus America Inc., Melville, NY, model IX50) and photographed using an Olympus® digital camera (Olympus America Inc., model DP71). Images were analyzed using ImageJ 1.42. The media lumen ratio was calculated from the media thickness/lumen diameter × 100.

### Measurement of collagen deposition

The thoracic aortae were dissected and processed as described above. Collagen staining was performed using Masson’s trichrome staining as described [16]. The stained sections were viewed in a blinded manner with an Olympus® inverted system microscope as described above. Percentage collagen positive area was analyzed using ImageJ 1.42.

### Measurement of vascular ROS production

To measure vascular reactive oxygen species production, 5 μm sections of the thoracic aorta were exposed to dihydroethidium (DHE), following the previously described method [11]. Fresh, unfixed aorta samples were placed in optimal cutting temperature (OCT) compound (Sakura Finetek USA Inc., Torrance, CA) and frozen at − 80 °C. Ring segments were cut into 30 μm sections using a cryostat (Bright Instrument Company, Huntingdon, Cambridgeshire, England; model OTF) and placed on a glass slide. Sections were incubated in PBS for 30 min at 37 °C, and then DHE (2 μm) was topically applied. Coverslips were applied, and the sections were further incubated at 37 °C in a light-protected humidified chamber for 30 min. Sections were then rinsed in PBS, and fluorescence was detected using a 585-nm filter using an Olympus® inverted system microscope (Olympus America Inc.; model DP71). Images were photographed using an Olympus® digital camera (Olympus America Inc., model DP71) and analyzed in a blinded manner using ImageJ 1.42.

### Statistical analysis

Data were analyzed by two-way analysis of variance followed by Tukey’s multiple comparisons post hoc test or the Student *t* test. Data values from the different experiments are expressed as the mean ± SEM. *P* < 0.05 was considered statistically significant.

## Results

### *Cyp1b1* gene disruption or castration in *Cyp1b1*^*+/+*^ and *Cyp1b1*^−/−^ mice reduced aortic responses to vasoconstrictor agents caused by Ang II infusion, which was restored by 6β-OHT

**Fig. 1:**
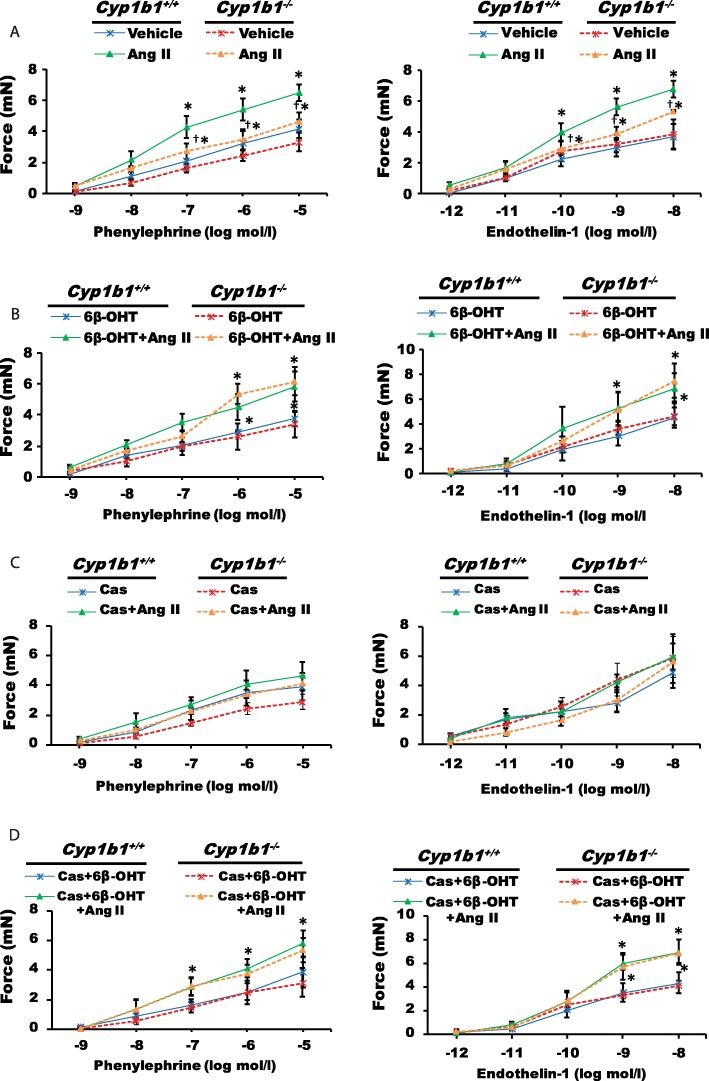
*Cyp1b1* gene disruption or castration minimized the increase in the aortic response to vasoconstrictor agents associated with angiotensin (Ang) II-induced hypertension, which was restored by 6β-hydroxytestosterone (6β-OHT). Intact or castrated *Cyp1b1*^*+/+*^ and *Cyp1b1*^*−/−*^ mice were infused with either Ang II (700 ng/kg/day) or vehicle for 14 days and given i.p. injections of 6β-OHT (15 μg/g b.w every third day) or its vehicle. Vascular reactivity was measured in the aorta as described above (**a**–**d**). The response of the aorta of intact or castrated *Cyp1b1*^*+/+*^ and *Cyp1b1*^*−/−*^ mice infused with Ang II and treated with 6β-OHT to increasing concentrations of phenylephrine (PE) and endothelin-1 (ET-1). *P < 0.05 vehicle, 6β-OHT, Cas+6β-OHT vs. corresponding values from Ang II-treated animals; ^†^*P* < 0.05 *Cyp1b1*^*+/+*^ Ang II vs. *Cyp1b1*^*−/−*^Ang II (*n* = 4–5 for all experiments, unpaired *t* test; data are expressed as mean ± SEM)

Ang II-induced hypertension was associated with an increased constriction of the isolated thoracic aortic rings (55% from *Cyp1b1*^*+/+*^ vehicle-treated group) (Fig. 1a) to maximal concentration of phenylephrine (PE) and (83% from *Cyp1b1*^*+/+*^ vehicle-treated group) to maximal concentration of endothelin-1 (ET-1); these increases were attenuated by *Cyp1b1* gene disruption (24% for PE, 52% for ET-1 compared to *Cyp1b1*^*+/+*^ vehicle-treated group), or castrated *Cyp1b1*^*+/+*^ and *Cyp1b1*^−/−^ mice (20% for PE and 21% and 16% for ET-1 compared to castrated *Cyp1b1*^*+/+*^ vehicle-treated group) (Fig. 1a, b). The 6β-OHT treatment restored the magnitude of aortic constriction to PE and ET-1 in both intact *Cyp1b1*^−/−^ (49% for PE and 94% for ET-1 compared to *Cyp1b1*^*+/+*^ + 6β-OHT) and castrated *Cyp1b1*^*+/+*^ and *Cyp1b1*^−/−^ mice (51% and 39% for PE, and 61% and 39% for ET-1 compared to castrated *Cyp1b1*^*+/+*^ 6β-OHT) infused with Ang II (Fig. 1b, c).

### *Cyp1b1* gene disruption or castration of *Cyp1b1*^*+/+*^ mice reduced endothelial dysfunction caused by Ang II infusion, which was restored by 6β-OHT

**Fig. 2:**
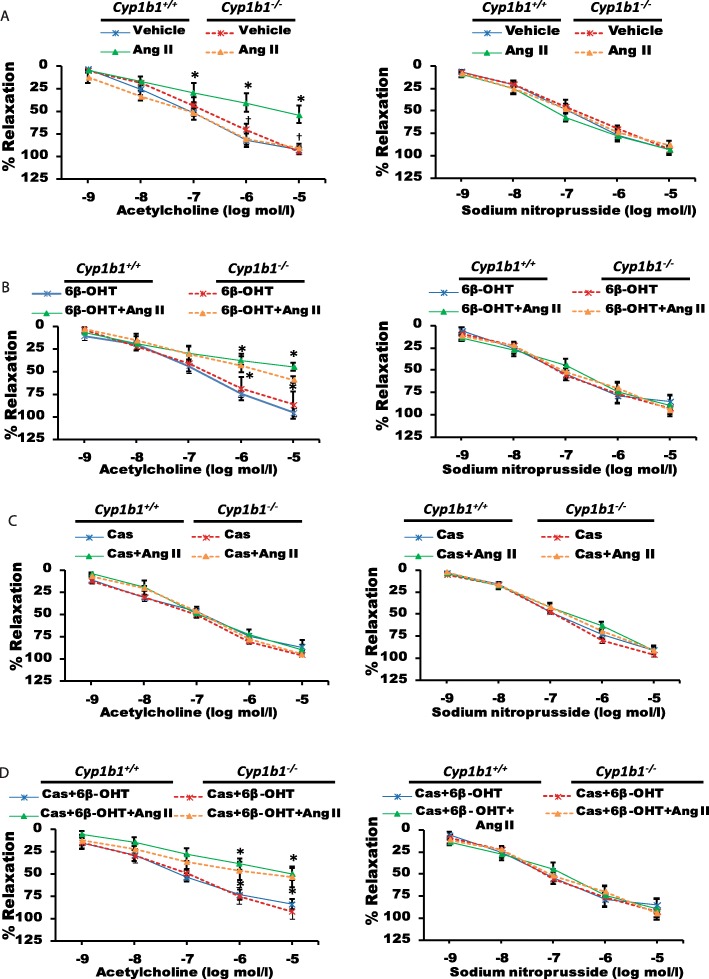
*Cyp1b1* gene disruption or castration reduced endothelial dysfunction associated with angiotensin (Ang) II-induced hypertension, which was restored by 6β-hydroxytestosterone 6β-OHT. Mice were infused with either Ang II (700 ng/kg/day) or vehicle for 14 days and given i.p. injections of 6β-OHT (15 μg/g b.w every third day) or its vehicle. Endothelial function was measured in the thoracic aorta, as described in the “Materials and methods” section (**a**–**d**). Vascular response to increasing concentrations of acetylcholine (ACh; endothelium-dependent relaxation) and sodium nitroprusside (SNP; endothelium-independent relaxation), respectively. *P < 0.05 vehicle, 6β-OHT, Cas+6β-OHT vs. corresponding values from Ang II-treated animals; ^†^*P* < 0.05 *Cyp1b1*^*+/+*^ Ang II vs. *Cyp1b1*^*−/−*^ Ang II (*n* = 4 for all experiments, unpaired *t* test; data are expressed as mean ± SEM)

Ang II infusion caused endothelial dysfunction in the aorta, as determined by the effect of ACh to induce maximal relaxation of the aorta pre-constricted with PE (54% *Cyp1b1*^*+/+*^ vehicle-treated group) (Fig. 2a). However, in the intact *Cyp1b1*^−/−^ or castrated *Cyp1b1*^*+/+*^ and *Cyp1b1*^−/−^ mice infused with Ang II, ACh- and SNP-induced relaxations of the aorta were not altered (Fig. 2a, b). The 6β-OHT treatment restored the effect of Ang II to cause endothelial dysfunction in both the intact *Cyp1b1*^*−/−*^ and castrated *Cyp1b1*^*+/+*^ and *Cyp1b1*^−/−^ mice (59%, 50%, 53%, respectively) as determined by the loss of relaxation of the aorta by ACh (88%) (Fig. 2c, d). SNP-induced vasodilation was not altered in these treatment groups.

### *Cyp1b1* gene disruption or castration in *Cyp1b1*^*+/+*^ and *Cyp1b1*^−/−^ mice reduced Ang II-induced aortic hypertrophy, which was restored by 6β-OHT

**Fig. 3:**
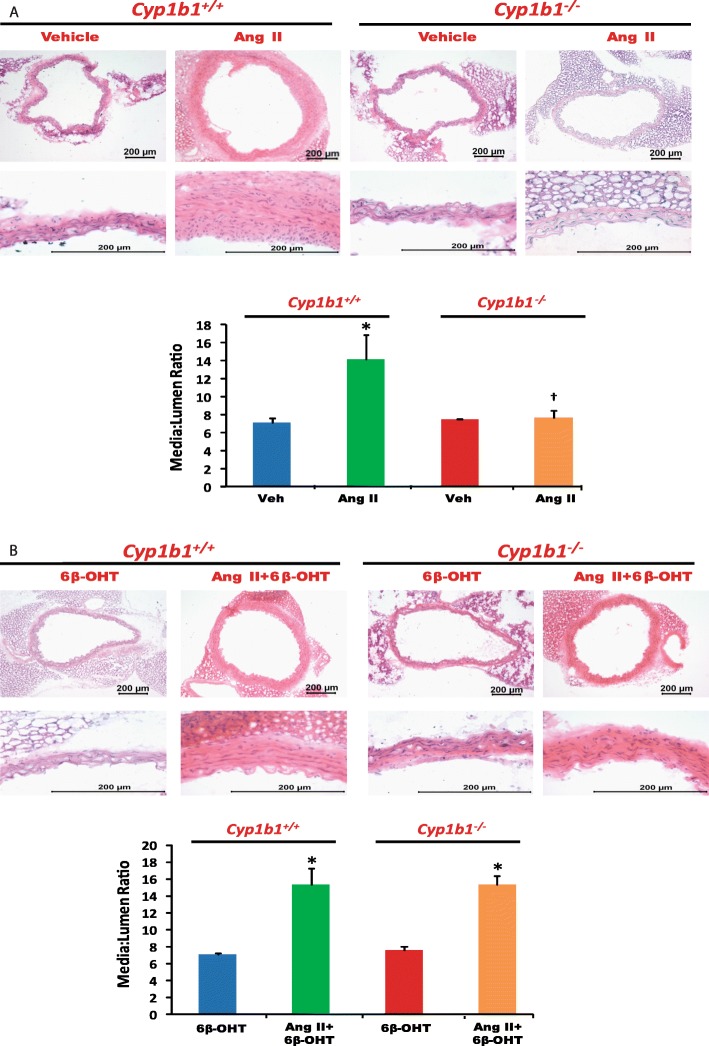
*Cyp1b1* gene disruption reduced vascular hypertrophy associated with angiotensin (Ang) II-induced hypertension, which was restored by 6β-hydroxytestosterone (6β-OHT). Mice were infused with vehicle or Ang II (700 ng/kg/day) for 14 days and injected with 6β-OHT (15 μg/g b.w every third day) as described in the “Materials and methods” section. After Ang II infusion, the aorta was removed and processed, H&E staining was performed, and the media/lumen ratio was calculated (**a**, **b**). *P < 0.05 vehicle, 6β-OHT vs. corresponding values from Ang II-treated animals; ^†^*P* < 0.05, *Cyp1b1*^*+/+*^ Ang II vs. *Cyp1b1*^*−/−*^ Ang II (*n* = 4–5 for all experiments; two-way ANOVA; data are expressed as mean ± SEM)

**Fig. 4:**
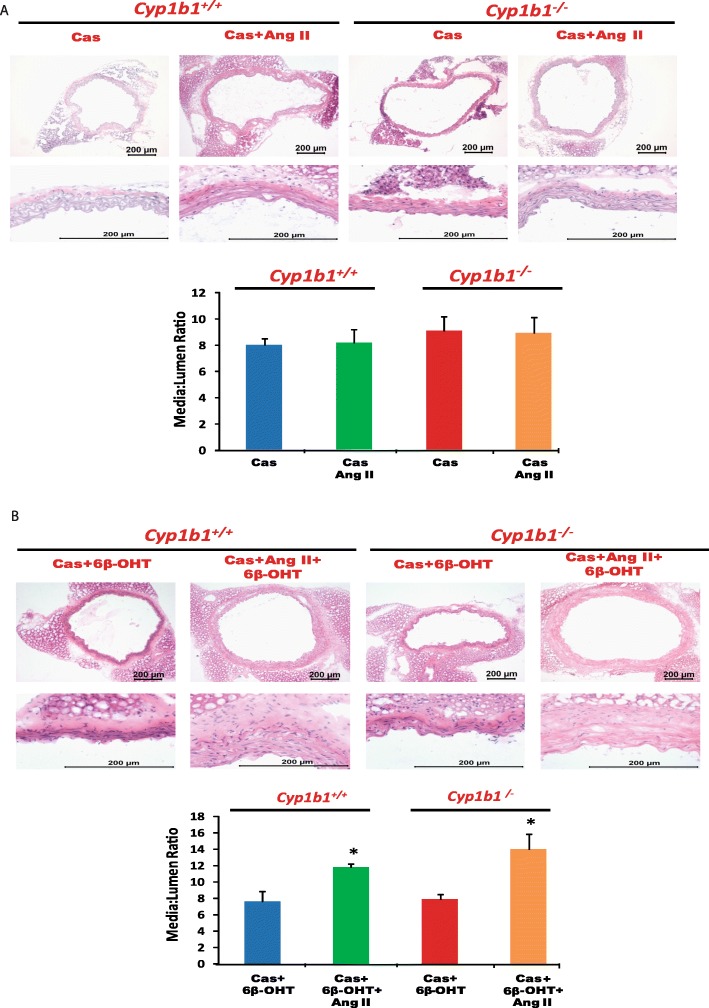
Castration reduced vascular hypertrophy associated with angiotensin (Ang) II-induced hypertension, which was restored by 6β-hydroxytestosterone (6β-OHT). Castrated mice were infused with vehicle or Ang II (700 ng/kg/day) for 14 days and injected with 6β-OHT (15 μg/g b.w every third day) as described in “Materials and methods” section. After Ang II infusion, the aorta was removed, H&E staining was performed, and the media/lumen ratio calculated (**a**, **b**). *P < 0.05 Cas+6β-OHT vs. corresponding values from Ang II-treated animals (*n* = 4–5 for all experiments; two-way ANOVA; data are expressed as mean ± SEM)

Ang II infusion in *Cyp1b1*^*+/+*^ mice caused vascular hypertrophy as determined by increased media to lumen ratio of the aorta (Fig. 3a). *Cyp1b1* gene disruption or castration in *Cyp1b1*^*+/+*^ and *Cyp1b1*^−/−^ mice reduced this ratio (Fig. 3a and Fig. 4a, respectively). The 6β-OHT treatment brought back the effect of Ang II to cause aortic hypertrophy in *Cyp1b1*^−/−^ and castrated *Cyp1b1*^*+/+*^ and *Cyp1b1*^−/−^ mice as indicated by increased media to lumen ratio of the aorta (Fig. 3b and Fig. 4b, respectively).

### *Cyp1b1* gene disruption or castration in *Cyp1b1*^*+/+*^ and *Cyp1b1*^−/−^ mice attenuated vascular fibrosis caused by Ang II, which was restored by 6β-OHT

**Fig. 5:**
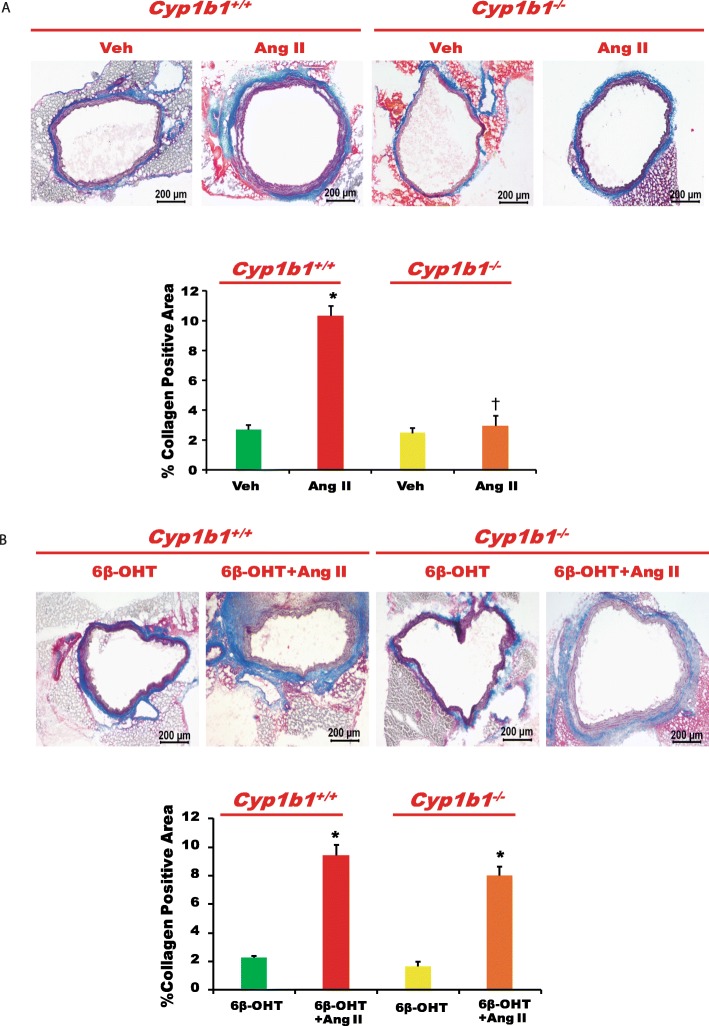
*Cyp1b1* gene disruption reduced vascular fibrosis associated with angiotensin (Ang) II-induced hypertension, which was restored by 6β-hydroxytestosterone (6β-OHT). Mice were infused with vehicle or Ang II (700 ng/kg/day) for 14 days and injected with 6β-OHT (15 μg/g b.w every third day) as described in the “Materials and methods” section. After Ang II infusion, the aorta was removed, processed, and stained with Masson’s trichrome to reveal collagen deposition (**a**, **b**). The percentage positive area for collagen staining was calculated. *P < 0.05 vehicle, 6β-OHT vs. corresponding values from Ang II-treated animals; ^†^*P* < 0.05, *Cyp1b1*^*+/+*^ Ang II vs. *Cyp1b1*^*−/−*^Ang II (*n* = 3 for all experiments; data are expressed as mean ± SEM)

**Fig. 6:**
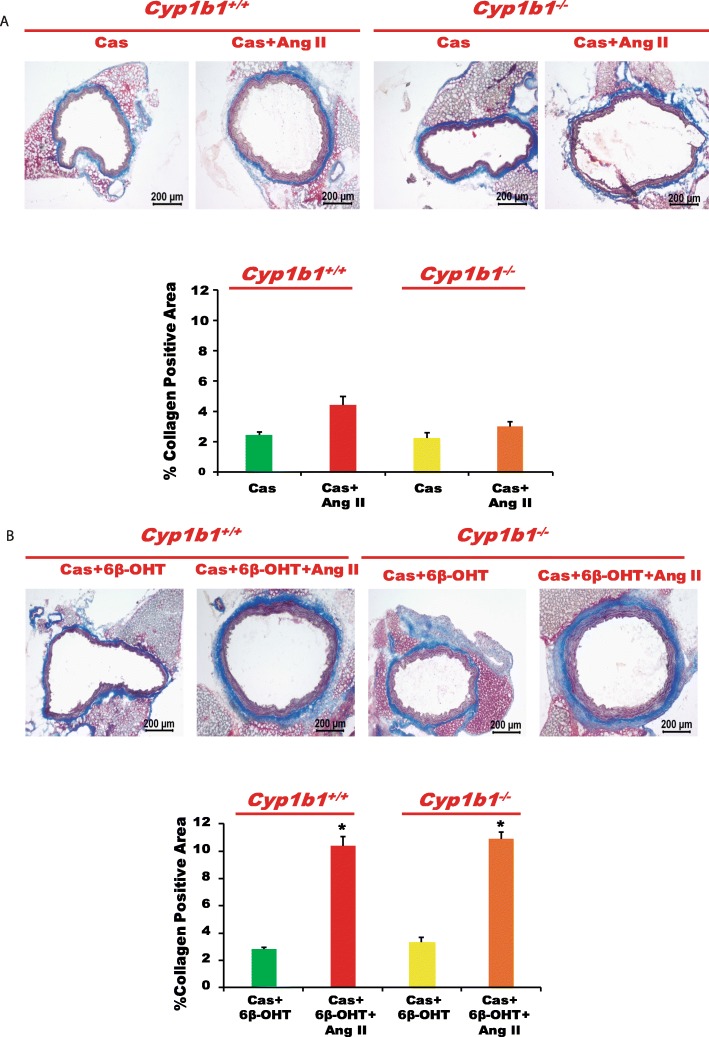
Castration (Cas) mitigated angiotensin (Ang) II-induced vascular fibrosis in *Cyp1b1*^*+/+*^ mice, which was restored by 6β-hydroxytestosterone (6β-OHT). *Cyp1b1*^*+/+*^ and *Cyp1b1*^*−/−*^ mice were castrated and infused with Ang II (700 ng/kg/day) and treated with 6β-OHT (15 μg/g b.w every third day). At the end of Ang II infusion, the aortas were removed, cut into sections, and stained with Masson’s trichrome staining for localization of collagen deposition (**a**, **b**). The percentage positive area for collagen staining was calculated. *P < 0.05 Cas + 6β-OHT vs. corresponding values from Ang II-treated animals (*n* = 3 for all experiments, two-way ANOVA; data are expressed as mean ± SEM)

Infusion of Ang II increased vascular fibrosis, as indicated by increased collagen staining in the aorta (Fig. 5a). *Cyp1b1* gene disruption or castration in *Cyp1b1*^*+/+*^ and *Cyp1b1*^−/−^ mice reduced collagen accumulation (Fig. 5a and Fig. 6a, respectively), which was restored by treatment with 6β-OHT (Fig. 5b and Fig. 6b, respectively).

### *Cyp1b1* gene disruption or castration in *Cyp1b1*^*+/+*^ and *Cyp1b1*^−/−^ mice infused with Ang II attenuated ROS generation, which was restored by 6β-OHT

**Fig. 7:**
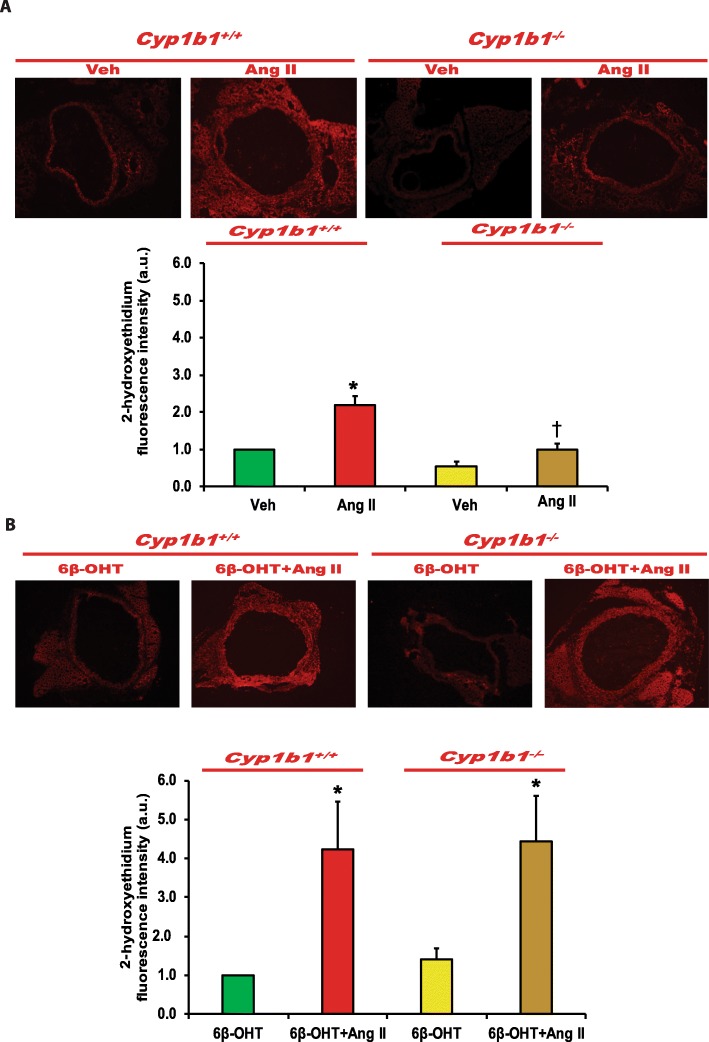
*Cyp1b1* gene disruption minimized angiotensin (Ang II)-induced superoxide production, which was reversed by 6β-hydroxytestosterone (6β-OHT). *Cyp1b1*^*+/+*^ and *Cyp1b1*^*−/−*^ mice were infused with vehicle or Ang II (700 ng/kg/day) (upper panel) and treated with 6β-OHT (15 μg/g b.w every third day) or 6β-OHT+Ang II (lower panel) for 14 days. Aortic superoxide production was determined by the fluorescence intensity of 2-hydoxyethidium (**a**, **b**). Photomicrographs are representative of the aorta from mice in each of the different treatment groups following incubation with dihydroethidium. The graph depicts the quantified data. *P < 0.05 vehicle, 6β-OHT vs. corresponding value from Ang II-treated animal; ^†^*P* < 0.05 *Cyp1b1*^*+/+*^ Ang II vs. *Cyp1b1*^*−/−*^ Ang II (*n* = 3 for all experiments, unpaired *t* test; data are expressed as mean ± SEM)

**Fig. 8:**
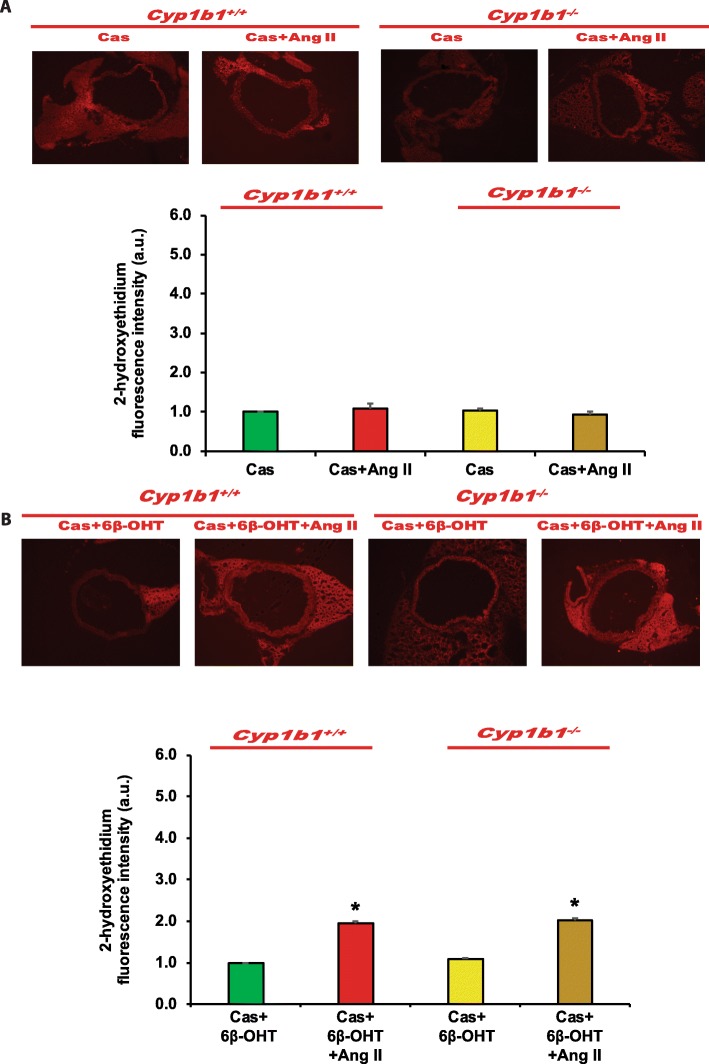
6β-Hydroxytestosterone (6β-OHT) restored angiotensin (Ang) II-induced superoxide production that was minimized in castrated (Cas) *Cyp1b1*^+/+^and *Cyp1b1*^*−/−*^ mice. *Cyp1b1*^*+/+*^ and *Cyp1b1*^*−/−*^ mice were castrated and then infused with vehicle or Ang II (700 ng/kg/day) (upper panel) and treated with 6β-OHT (15 μg/g b.w every third day) or 6β-OHT+Ang II (lower panel) for 14 days. **a** Aortic superoxide production was determined by the fluorescence intensity of 2-hydoxyethidium. **a**, **b** Photomicrographs are representative of the aorta from mice in each of the different treatment groups following incubation with dihydroethidium. **b** The graph depicts the quantified data. *P < 0.05 vehicle vs. corresponding values from Ang II-treated animals (*n* = 3 for all experiments, unpaired *t* test, and the data are expressed as mean ± SEM)

Ang II infusion increased vascular ROS production as indicated by increased 2-hydroxyethidium fluorescence in the aorta of *Cyp1b1*^*+/+*^, but not in the intact *Cyp1b1*^−/−^ or castrated *Cyp1b1*^*+/+*^ and *Cyp1b1*^−/−^ mice (Fig. 7a and Fig. 8a, respectively). Treatment with 6β-OHT restored the ability of Ang II to increase ROS production in the aorta of intact *Cyp1b1*^−/−^ or castrated *Cyp1b1*^*+/+*^ and *Cyp1b1*^−/−^ mice (Fig 7b and Fig. 8b, respectively).

### Androgen receptor antagonist flutamide reduced increase in SBP and vascular hypertrophy in castrated *Cyp1b1*^*+/+*^ mice infused with Ang II and treated with 6β-OHT

**Fig. 9:**
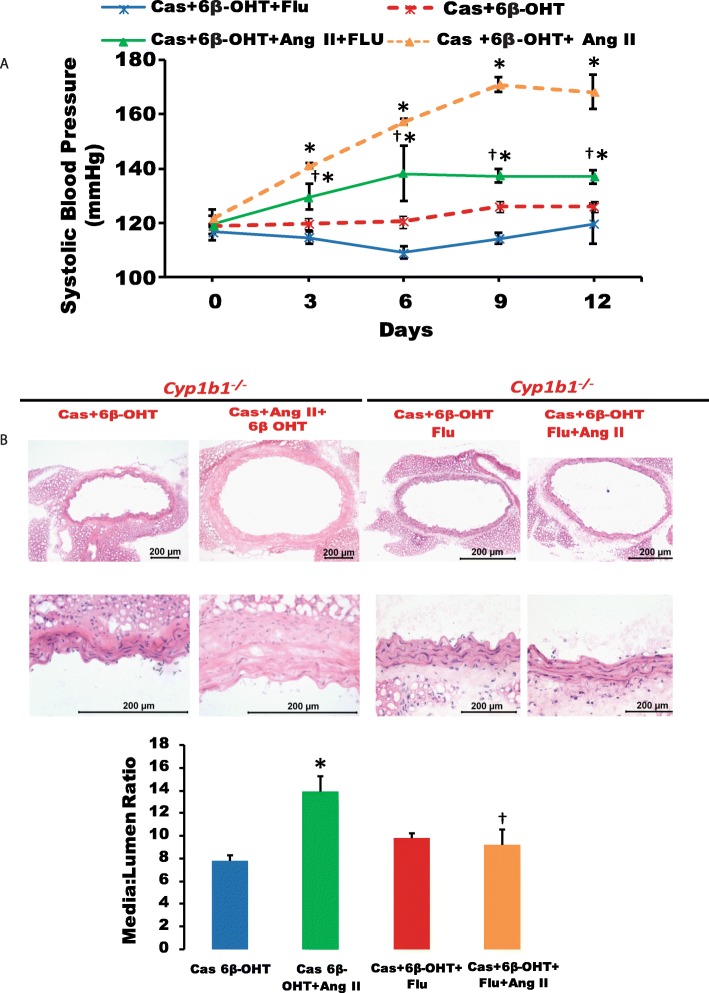
Flutamide reduced systolic blood pressure and vascular hypertrophy in castrated mice treated with 6β-hydroxytestosterone (6β-OHT). Castrated *Cyp1b1*^*+/+*^ mice were infused with vehicle or angiotensin (Ang) II (700 ng/kg/day) and treated with 6β-OHT(15 μg/g b.w every third day) and flutamide (FLU) (8 mg/kg every day), and blood pressure was measured (**a**). H&E staining was performed to determine vascular hypertrophy, and the media to lumen ratio was calculated (**b**). The graph depicts the quantified data. *P < 0.05 Cas+6β-OHT, Cas+6β-OHT+FLU vs. corresponding values from Ang II-treated animals; ^†^*P* < 0.05 Cas+ 6β-OHT+FLU+Ang II vs. Cas+ 6β-OHT+Ang II (*n* = 5 for all experiments, two-way ANOVA; data are expressed as mean ± SEM)

Castration in *Cyp1b1*^*+/+*^ mice reduced the Ang II-induced increase observed in SBP and aortic hypertrophy, which was restored by 6β-OHT (Fig. 9a, b, respectively). Treatment with flutamide reduced this increase in SBP and vascular hypertrophy (*P* < 0.05) (Fig. 9a, b, respectively).

## Discussion

The main findings of this study are that 6β-OHT, a metabolite of testosterone generated by CYP1B1, contributes to the effects of Ang II to (1) increased vascular reactivity to PE and ET-1, (2) endothelial dysfunction, (3) vascular hypertrophy, (4) vascular fibrosis, and (5) oxidative stress. Previously, we reported that *Cyp1b1* gene disruption or chemical inhibition of CYP1B1 activity minimized the Ang II-induced increase in vascular reactivity to vasoconstrictor agents, increased vascular ROS production, and endothelial dysfunction [11]. Moreover, we showed that Ang II stimulates the production of 6β-OHT in *Cyp1b1*^*+/+*^, but not *Cyp1b1*^*−/−*^ mice, and it is required (i.e., acts as a permissive factor) for Ang II-induced hypertension and associated cardiac remodeling and renal dysfunction [16, 17]. The current study assessed the contribution of 6β-OHT to the action of Ang II on alterations in the vascular function, endothelial dysfunction, hypertrophy, fibrosis, and ROS production. The results revealed that Ang II infusion for 14 days increased the response of thoracic aorta to PE and ET-1 in *Cyp1b1*^*+/+*^ mice, and these effects of Ang II were minimized in *Cyp1b1*^*−/−*^ mice, thus confirming our previous results [11]. Administration of 6β-OHT to *Cyp1b1*^*−/−*^ mice that do not generate this testosterone metabolite [16] restored the increase in the response of the thoracic aorta to PE and ET-1 in Ang II-infused intact *Cyp1b1*^−/−^ or castrated *Cyp1b1*^+/+^ and *Cyp1b1*^−/−^ mice. Therefore, it appears that 6β-OHT treatment alone, which did not alter the vascular response to PE and ET-1, is necessary for the expression of the increase in vascular reactivity caused by Ang II infusion in the male mice.

Ang II infusion also caused endothelial dysfunction as indicated by the attenuation of relaxation to ACh, but not to SNP in the aorta of *Cyp1b1*^*+/+*^ male mice. *Cyp1b1* gene disruption minimized the effect of Ang II [16]. In the present study, we found that in Ang II-infused *Cyp1b1*^*−/−*^ mice, administration of 6β-OHT caused endothelial dysfunction in the aorta. Infusion of Ang II also produced vascular hypertrophy as indicated by the increased media to lumen ratio of the aorta in *Cyp1b1*^+/+^ mice; this effect of Ang II was abrogated in *Cyp1b1*^*−/−*^ mice. However, 6β-OHT restored the effect of Ang II to cause vascular hypertrophy in *Cyp1b1*^*−/−*^mice. 6β-OHT also mediates the effect of Ang II in causing aortic fibrosis, because the Ang II-induced collagen accumulation that was abrogated in *Cyp1b1*^*−/−*^mice was significantly restored by 6β-OHT. Further, support for the role of 6β-OHT in the action of Ang II to increase vascular reactivity, endothelial dysfunction, vascular hypertrophy, and fibrosis was obtained in castrated mice. Castration attenuated the increase in vascular reactivity, endothelial dysfunction, vascular hypertrophy, and fibrosis in Ang II-infused *Cyp1b1*^*+/+*^ mice. However, concurrent administration of 6β-OHT restored the effects of Ang II to increase aortic reactivity to PE and ET-1 and cause endothelial dysfunction, hypertrophy, and fibrosis in castrated *Cyp1b1*^*+/+*^ mice. The mechanism by which 6β-OHT mediates the effects of Ang II to increase vascular reactivity and to cause endothelial dysfunction, hypertrophy, and aortic fibrosis in intact *Cyp1b1*^*−/−*^ and castrated *Cyp1b1*^*+/+*^ and *Cyp1b1*^*−/−*^ mice could be the consequence of the restoration of Ang II-induced increase in BP [16]. However, further studies in vitro and in vivo using hydralazine, a direct vasodilator, are required to establish the BP-dependent and BP-independent mechanism by which 6β-OHT mediates the vascular effects of Ang II.

Ang II increases vascular ROS production in rats and mice, and renal cortex in SHR [20–22]. *Cyp1b1* gene disruption and inhibition of its activity decrease vascular and renal oxidative stress in rats and mice [11–15]. Moreover, 6β-OHT was shown to mediate Ang II-induced increases in cardiac and renal oxidative stress [16–17]. Our finding that administration of 6β-OHT to the intact *Cyp1b1*^*−/−*^ or castrated *Cyp1b1*^*+/+*^ and *Cyp1b1*^*−/−*^ mice infused with Ang II increased ROS production suggests that 6β-OHT is required for the restoration of the effect of Ang II to increase oxidative stress. The endothelial dysfunction is, in part, attributed to the result of inactivation of NO by ROS [23]. Therefore, it appears that by mediating Ang II-induced ROS production in the aorta, 6β-OHT causes endothelial dysfunction. The increase in ROS production by Ang II infusion could result in increased vascular reactivity and hypertrophy through activation of ERK1/2 and p38 MAPK, which are known to mediate Ang II-induced hypertrophy in cultured VSMCs [24, 25]. Previously, we reported that Ang II increased aortic ERK1/2 and p38 MAPK activity and that these effects were attenuated by treatment with the CYP1B1 inhibitor 2,3′,4,5′-tetramethoxystilbene [11]. Moreover, 2,3′,4,5′-tetramethoxystilbene in cultured rat VSMCs or cells transduced with adenovirus CYP1B1 short hairpin RNA, Ang II- and arachidonic acid-induced increase in ERK1/2 and p38 MAPK activities were inhibited without alterations in the expression of Ang II type 1 receptor or its coupling to G proteins [26].

CYP1B1 gene disruption did not alter the expression of AT1 receptor, ACE, or Mas receptor in the heart or kidney of mice infused with Ang II [16, 17]. However, the expression of AT1 receptor and ACE in the kidney was reduced by castration in *Cyp1b1*^*+/+*^ and *Cyp1b1*^*−/−*^ mice, and it was reversed and enhanced by treatment with 6β-OHT. Whether 6β-OHT increases the aortic expression of AT1 receptor and ACE that contributes to the effect of Ang II to increase vascular reactivity and cause endothelial dysfunction, hypertrophy, aortic fibrosis, and ROS production remains to be determined.

Administration of the androgen receptor blocker flutamide either peripherally or centrally reduced the increase in BP observed in Ang II-infused mice and in transgenic hypertensive TGR(mREN2) rats (TGR) harboring the murine Ren-2 gene [9, 27–28]. Flutamide also prevented endothelial dysfunction and an increase in vascular reactivity in diabetic Zucker rats and Ang II-infused mice [29–32]. In the present study, the effect of 6β-OHT to restore the Ang II-induced increase in BP and aortic hypertrophy that was reduced by castration in *Cyp1b1*^*+/+*^ mice was inhibited by flutamide. This is the first evidence suggesting that 6β-OHT contributes to the effect of Ang II on BP and hypertrophy via the androgen receptor. However, further studies are required to determine if it involves the DNA- or non-DNA-dependent androgen receptor or G-protein coupled androgen receptor (GPRC6A) [33]. Moreover, the effect of flutamide on the 6β-OH-mediated effect of Ang II to increase vascular reactivity and produce endothelial dysfunction also needs to be examined. Testosterone has been reported to downregulate the expression of the AT2 receptor via the androgen receptor-mediated ERK1/2 MAP kinase pathway in rat aorta [34]. Whether 6β-OHT mediates the vascular effects of Ang II by downregulating AT2 receptors in the mice aorta remains to be explored.

## Perspectives and significance

This study provides evidence that 6β-OHT, a metabolite of testosterone generated by CYP1B1, acts as a permissive factor that contributes to the effects of Ang II to increase vascular reactivity; cause endothelial dysfunction, vascular hypertrophy, and fibrosis; and increase oxidative stress in male mice. Moreover, the effect of 6β-OHT on Ang II-induced increases in BP and aortic hypertrophy is mediated by the androgen receptor. In contrast to male mice, we have shown that Ang II produces a lower increase in BP in *Cyp1b1*^*+/+*^ as compared to *Cyp1b1*^*−/−*^ female mice [11]. This diminished ability of Ang II to increase BP in female *Cyp1b1*^*+/+*^ mice is associated with decreased cardiac and vascular smooth muscle remodeling, reduced endothelial dysfunction, and decreased vascular reactivity to PE and ET-1 [11]. Protection against the increase in vascular reactivity, endothelial dysfunction, and ROS production associated with hypertension in female mice was found to be due to the CYP1B1-17β-estradiol generated metabolite 2-methoxyestradiol [13]. Therefore, CYP1B1 could serve as a novel target for developing agents that inhibit CYP1B1 for treating the increased vascular reactivity, endothelial dysfunction, vascular hypertrophy, fibrosis, and ROS production associated with Ang II- and testosterone-dependent hypertension in males, but inhibitors of CYP1B1 could be detrimental in treating vascular changes associated with hypertension in females.

## Data Availability

The data supporting the findings of our manuscript are available upon reasonable request by email to the corresponding author.
